# Arsenic Uptake and Accumulation Mechanisms in Rice Species

**DOI:** 10.3390/plants9020129

**Published:** 2020-01-21

**Authors:** Tayebeh Abedi, Amin Mojiri

**Affiliations:** 1 Umea Plant Science Centre, Department of Forest Genetics and Plant Physiology, Swedish University of Agricultural Sciences, 90183 Umea, Sweden; 2 Department of Civil and Environmental Engineering, Graduate School of Civil Engineering, Hiroshima University, 1-4-1 Kagamiyama, Higashihiroshima 739-8527, Japan; amojiri@hiroshima-u.ac.jp

**Keywords:** arsenic, detoxification, rice, toxicology

## Abstract

Rice consumption is a source of arsenic (As) exposure, which poses serious health risks. In this study, the accumulation of As in rice was studied. Research shows that As accumulation in rice in Taiwan and Bangladesh is higher than that in other countries. In addition, the critical factors influencing the uptake of As into rice crops are defined. Furthermore, determining the feasibility of using effective ways to reduce the accumulation of As in rice was studied. AsV and AsIII are transported to the root through phosphate transporters and nodulin 26-like intrinsic channels. The silicic acid transporter may have a vital role in the entry of methylated As, dimethylarsinic acid (DMA) and monomethylarsonic acid (MMA), into the root. Amongst As species, DMA(V) is particularly mobile in plants and can easily transfer from root to shoot. The *OsPTR7* gene has a key role in moving DMA in the xylem or phloem. Soil properties can affect the uptake of As by plants. An increase in organic matter and in the concentrations of sulphur, iron, and manganese reduces the uptake of As by plants. Amongst the agronomic strategies in diminishing the uptake and accumulation of As in rice, using microalgae and bacteria is the most efficient.

## 1. Introduction

Rice (*Oryza sativa* L.) provides food for more than three billion people [1]. Approximately 90% of rice production and consumption is reported in Asia [2]. By 2050, the production of rice should rise by 60%–70% to meet the requirements of the predicted population growth in Asia [3]. Rice can be cultivated in many regions of the world because of its resourcefulness and diversity. Two species, *Oryza glaberrima* Steud. (in Africa) and *Oryza sativa* L. (in Asia), are commonly cultivated [4]. However, rice consumption can pose problems because of the arsenic (As) accumulation in rice and thus serves a vital source of As exposure in humans [5].

As is the 20th abundant component on the Earth’s crust. However, As is a toxic metalloid and is remarked as a considerable global groundwater contaminant, affecting certain rivers and deltas in East and South Asia and in South American countries [6]. Based on the Agency for Toxic Substances and Disease Registry list 2017, As is amongst the most hazardous materials that could be poisonous to humans. Approximately 200 million people in around 70 countries have been exposed to this metalloid [7].

As enters agricultural lands and the environment via natural sources, such as rocks, As-enriched minerals, forest fires, volcanoes and anthropogenic sources (e.g., mining, herbicides, phosphate fertilisers, smelting, industrial processes, coal combustion and timber preservatives) [8]. The average amount of As in agricultural fields that receive As-comprising pesticides and defoliants ranges from 5 mg/kg to 2553 mg/kg [9]. The management of paddy soils for the wet cultivation of rice involves different cycles of submerged and dry days, which cause alternating oxidising and reducing processes in soil. Under this condition in paddy soil, As is reduced to arsenite (AsIII) with high toxicity and mobility in flooded soil, AsIII may then be taken up by rice [10]. Evidence shows considerable As toxicity from the utilisation of rice and rice-based products, especially those consumed as a staple dietary source [11]. Accumulation of As in rice may be decreased by amending cultural practices. Hence, the current work focused on the mechanisms of uptake and accumulation of As in rice and the effective factors which reduce As uptake by rice crops. The results are expected to enrich our understanding of the uptake, transport and distribution of As in rice.

## 2. Arsenic Uptake and Transport by Rice Plants

Rice is more seriously affected by As pollution than other crop plants. Its cultivation is carried out in flooded conditions, which lead to the reduction conditions [12]. As may be found in the environment, organic and inorganic AsIII and arsenate (AsV) are the dominant As species that reduce paddy soil conditions, followed by methylated As species. Moreover, plant roots selectively may uptake specific As forms [13]. Table 1 shows the reported concentrations of As in rice around the world. All As species can be transferred through the plant cell via specific transporter proteins [14]. The genes involved in transporting As in rice are shown in Table 2.

### 2.1. Uptake of Inorganic Arsenic

Inorganic As, AsIII and AsV, is more toxic than organic As, dimethylarsinic acid (DMA) and monomethylarsonic acid (MMA) [18]. Inorganic As is the dominant form of As in soil and groundwater. Under aerobic conditions in soil, AsV dominates, whereas, in submerged conditions, AsIII is the dominant species [40].

AsV may generally enter the roots of rice crops through phosphate transporters (PHTs), primarily PHT1 (phosphate transporter1)-type transporters [41] during the regulation of inorganic phosphorous (Pi) [42]. A total of 13 PHTs from the PHT1 family in rice (*Oryza sativa*) have been found to mediate Pi uptake and transport [43] through the highly efficient silicon (Si) uptake pathway.

Furthermore, AsIII may enter by the nodulin 26-like intrinsic (NIPs) aquaporin channels accompanied by silicic acid and ammonia [42]. NIP proteins are one of the major intrinsic proteins which comprise the family of important membrane channel proteins [44]. NIPs are categorised into three main groups, namely, NIP-I, NIP-II and NIP-III, with regard to the common substrate selectivity and consistency of amino acid composition [45]. The NIP-II group (such as OsNIP3;1, AtNIP5;1, AtNIP6;1 and ZmNIP3;1) has been found to be vital for the uptake and transporting of boron in several plants [44]. NIP-III group members (such as OsNIP2;1, OsNIP2;2, HvNIP2;1, HvNIP2;2 and CmNIP2;1) are revealed to be vital for the effectual uptake and translocation of Si [46]. A family of 10 NIP proteins is found in rice. Bienert et al. [47] reported that NIPs in *Oryza sativa* L., OsNIP2;1 (Lsi1) and OsNIP3;2 (Lsi2) are capable of facilitating an influx of AsIII into rice root cells.

### 2.2. Uptake of Organic Arsenic

Methylated As species, namely, MMA (CH_3_AsO(OH)_2_) and DMA ((CH_3_)_2_AsOOH), might be present in soil due to microbial actions or past usage of methylated As compounds, cacodylic acid or sodium salt of MMA and DMA as pesticides [42]. Microorganisms in soil may convert As species from AsV to AsIII and further to MMA and DMA. Suriyagoda et al. [48] stated that DMA and MMA might be taken up by the silicic acid transporter Lsi1. Plant roots are capable of taking up DMA and MMA, but the amounts of uptake are lower than those of inorganic As species and diminish with increasing numbers of methyl groups [49]. MMA(V) is partially reduced to trivalent MMA(III) in rice roots, but only MMA(V) is translocated to shoots [50]. DMA(V) is mobile in plants and may easily transfer from root to shoot. Muehe et al. [51] explained that methylated As is taken up gradually into rice relative to inorganic species but that it is freely translocated to grains.

### 2.3. Arsenic Species Translocation from Root to Shoot

The average translocation factor for As is around 0.8, which is higher than the other crops, such as barley (0.2) and wheat (0.1) [52].

As mentioned previously, the Si transporter OsNIP2;1 (Lsi1) is responsible for AsIII uptake, and Lsi2 drives AsIII efflux from rice root cells to the xylem [53]. Lsi1 is located in the distal side of the plasma membranes in exodermal and endodermal cells and is in charge of the influx of AsIII, whilst Lsi2 is located in the proximal sides of the same root cells and is in charge of the efflux of AsIII [54]. In other words, the synergy of Lsi1 and Lsi2 transports Si and AsIII into root cells. Si and AsIII in the xylem vessel are transported to the shoot via transpiration flow by Lsi1 and Lsi2 first and then by Lsi6, which is localised in the xylem parenchyma cells of leaves [55]. Detmann et al. [56] stated that the *Lsi6* gene plays a vital role in the distribution of Si, or maybe AsIII, in rice shoot.

In rice, 13 Pi transporter genes (*OsPT*) can transport AsV to the root. Amongst these genes, *OsPT1, OsPT2, OsPT4, OsPT6, OsPT8, OsPT9* and *OsPT10* have a role in P uptake and translocation in rice. Certain Pi transporters also mediate AsV uptake [57]. Amongst these OsPTs, *OsPT1* is thoroughly stated in roots and is a main regulator of Pi. *OsPT2* has a vital role in Pi transferring from the root to the shoot [58]. AsV may be reduced to AsIII inside the rice root, and AsIII may then enter the xylem via a silicic acid/AsIII effluxer [59]. Chen et al. [60] explained that AsV may be quickly reduced to AsIII in plant cells by high As content 1 (HAC1) AsV reductases. Chao et al. [61] reported that HAC1 is vital in the reduction of AsV activity in the outer layer of the root (epidermis) and the inner layer adjacent to the xylem (pericycle).

Mitra et al. [14] expressed that DMA and MMA enter the NIP protein. However, AsIII is more capably taken up by roots than MMA and DMA. Zhao et al. [49] stated that the transferring from roots to shoots commonly rises with the rising quantity of methyl groups in As. DMA is extremely mobile throughout the xylem and phloem in rice. Tang et al. [62] reported that *OsPTR7* may interact in the transferring of DMA in the phloem or xylem. High mobility of DMA occurs in the moving from roots to shoots and from leaves to grain.

### 2.4. Phloem and Xylem-Derived Pathways of As Species and As Loading in Grains

As may be transferred from roots to shoots through the xylem [48]. Phloem transportation is probably responsible for 54%, 56%, 100% and 89% of AsIII, AsV, MMA(V) and DMA(V) translocation into rice grains, respectively. In the phloem, organic arsenics are more transportable than inorganic arsenics [63]. Moreover, AsIII is transported to rice grains principally through the phloem pathway, whereas DMA is translocated to rice grains through the xylem and phloem pathways [62]. In arabidopsis, Duan et al. [64] reported that AtINT2 and AtINT4 (inositol transporters) may have a role in AsIII entering into the phloem and in adjusting the accumulation of As(III) in seeds. Hence, similar transporters in rice plants may be responsible for As(III) transport. Furthermore, *OsPTR7* has a role in the long-distance transferring of DMA and in the accumulation of DMA in rice grains [62].

### 2.5. Phytotoxicity of Arsenic and Arsenic Detoxification Mechanism in Rice Plants

As is extremely phytotoxic to plants as it diminishes plant growth and crop yield [18]. Several rice varieties were subjected to AsIII and AsV by Shakoor et al. [67]. Seed germination was slightly limited at 0.5 and 1 mg·L^−1^, and a diminishing of around 10% in germination was detected at 2 mg·L^−1^. The growth of root was limited by 20% at 0.5 mg·L^−1^ of AsV. In addition, AsV was found to be more toxic than AsIII. The dangerous biochemical impact of As at the subcellular level is the production of reactive oxygen species (ROSs), such as hydroxyl radical (OH), superoxide radical (O_2_^−^) and hydrogen peroxide. ROSs are hazardous for plant metabolism and may lead to damage to macromolecules [68].

As previously mentioned, AsV is reduced to AsIII in plants. In addition, AsIII efflux to the external medium is a vital way of As detoxification in plants. Inside plant cells, AsIII may be detoxified by complexation with phytochelatins (PCs), followed by the accumulation of AsIII–PC complexes in vacuoles through OsABCC1 transporters [69]. OsABCC1 is one of the ATP-binding cassette (ABC) transporters. ABC transporter proteins play roles in the translocation of a broad range of substances within membranes using energy from ATP hydrolysis [70]. Song et al. [66] stated that OsABCC1 plays a vital role in the detoxification and decreasing As in rice grains.

HAC1 contributes to the defence against As in plants [71] and is essential for the efflux of AsIII from roots for AsV detoxification [72]. In rice, OsHAC1;1, OsHAC1;2 and OsHAC4 function as AsV reductase. Moreover, glutaredoxin possesses AsV reductase enzyme activity in maintaining the glutathione (GSH) pool and assists in AsIII efflux [73].

Brinke et al. [74] stated that with rising AsIII concentrations, the importance of the term ‘response to stress’ is replaced by the detoxification ways ‘glutathione biosynthesis’, which is related to the term ‘oxidation reduction’. GSH is applied as an electron donor by dehydroascorbate reductase to reconvert dehydroascorbate to ascorbate. GSH disulphide is the oxidised form of GSH, which may be reprocessed to GSH by glutathione reductase via reduced nicotinamide adenine dinucleotide phosphate. Hence, these different components of the ascorbate–GSH cycle may have a vital character in protecting cells against oxidative damage resulted by As toxicity [75].

After the reduction of AsV to AsIII, further mechanisms of detoxification arise in the vacuole via vacuolar sequestration. AsIII chelates with sulfhydryl (–SH)-rich protein and arranges a complex that is separated by vacuolar transporters (PCs). In rice, two phytochelatin synthase enzymes have been testified, comprising OsPCS1 and OsPCS2 [73].

## 3. Effects of Different Factors on Reducing Arsenic Uptake by Plants

Certain factors, such as pH, soil texture, organic matter (OM) and sulphide concentrations, may affect As uptake by plants [76]. Soil texture may affect As mobility due to differences in charges on the soil surface, which controls the adsorption and desorption procedures in soil. Soils with high amounts of clay have a higher As retention potential than coarse-textured soils. Reports also indicated that As uptake and concentrations are higher in plants grown in loamy sand than in plants grown in silty clay loam soils [77]. Moreover, As is five times more toxic in sand and loam than in clay soil, and its available form is a vital factor related to phytotoxicity [78].

### 3.1. Soil pH

pH is an important factor affecting As uptake [79]. An increase in pH commonly results in the mobilisation of As in soil. In general, an increase in soil pH results in a release of anions from within their exchange positions, along with AsV and AsIII [80]. Tu and Ma [79] reported that redox potential and pH influence As species. For example, under oxidising situations at pH < 6.9, H_2_AsO_4_^−^ becomes the primary species, and at a high pH, HAsO_4_^2−^ is dominant. High soil pH (generally pH 8.5) increases the negative surface charges, such as hydroxyl ions, thereby facilitating the desorption of As from Fe oxides and the resulting mobilisation of As in the root area; these conditions, in turn, increase As accumulation in plants [14].

### 3.2. Soil Organic Matter

OM in soil may influence the mobility and bioavailability of As over redox reactions, anions (phosphate, DOC and silicate), As–OM complexation and competitive adsorption [81]. OM may also affect plant growth and As accumulation in rice plants. OM, theoretically, insolubilises As over certain mechanisms, such as the binding of As with phenolic OH, carboxylate and sulfhydryl groups with/without ternary complexes [82]. Norton et al. [83] stated that OM is important in the mobilisation of As from paddy fields because microbes utilising OM consume oxygen that results in a reduction in redox potential, which leads to As dissolution from FeOOH. Syu et al. [81] explained that the characteristics of soils and OMs should be considered before using OM amendments to As-polluted soils. The use of OM to As-polluted soils may exert different effects on the As accumulation and growth of rice plants [81]. For example, biochar may improve As reduction and release in flooded paddy soils [84], but augmenting farmyard manure to soils with high amounts of As leads to a reduction of plant growth [83]. Norton et al. [83] stated that OM may also play two other roles in As availability in soils: by desorbing As species from soil surface exchange sites and complexing As species with dissolved organic matter (DOM).

### 3.3. Concentration of Nitrogen, Phosphorous and Sulphate in Soil

In rice soils, the main form of nitrogen (N) is ammonium, whilst nitrate concentration is less than 10 μM. Rice roots discharge oxygen into the rhizosphere, thereby causing ammonium nitrification by microbes near the root surface [85]. The procedure of Fe redox cycling may be influenced by N cycling. The coupled NO_3_^−^ reduction and Fe(II) oxidation can diminish As in paddy environments [63].

As previously mentioned, rice crops take up AsV via phosphate transporters [57]. Phosphate and arsenate are analogues and may compete for the same sorption sites on soil particles. The adding of phosphate commonly has two consequences: (i) raised downward move of As resulting in increased leaching from the topsoil and (ii) enhanced accessibility of As in the soil solution. AsV also acts as a phosphate analogue with respect to transport across the root plasma membrane [86]. Pigna et al. [87] reported that As toxicity in crops may be prevalent in situations where As pollution coexists with low available P.

Sulphur (S) is an element that interacts toughly with As, particularly under reducing conditions; the reduced forms of S can make a binding with As(III) [88]. Srivastava et al. [89] stated that S is important for plant growth as it regulates As tolerance over complexation of As by S-containing ligands (glutathione [GSH; γ-Glu-Cys-Gly] and PCs [GSH oligomers]). Zhang et al. [90] reported a reduction in translocating As from roots to shoots in high sulphate-pretreated rice plants.

### 3.4. Concentration of Iron and Manganese in Soil

Anwar et al. [9] expressed that Fe and Mn-rich compounds, such as goethite, ferruginous smectites, nontronite, pyrolusite and birnessite, absorb large amounts of As(V). Hence, As mobility may be low. A coating of Fe hydroxides/oxides identified as iron plaque, is normally formed on the roots of aquatic plant species. Iron plaque is the result of the oxidation of roots by releasing oxygen and oxidants into the rhizosphere [91,92]. Iron plaque also restrains the uptake of As by plants, possibly due to its adsorption or co-precipitation procedures [93]. Yu et al. [94] reported a negative correlation between As in rice grains and amorphous Fe oxide-bound As in soil and specified that amorphous Fe oxides might play a role as a barrier for As uptake by the plant. Liu et al. [91] expressed that Mn and Fe plaque can reduce the uptake of As in rice seedlings.

## 4. Agronomic Methods for Reducing Uptake and Accumulation of Arsenic by Plants

Awasthi et al. [12] stated three key plans to decrease As uptake by rice: (1) agronomic practices; (2) transforming the transporters involved in uptake; and (3) influencing the mobility of As in developing the synthesis of chelators.

For the agronomic strategy, researchers have tried to reduce As uptake by rice using different mitigation methods (Table 3). Several strategies are being practiced to mitigate As pollution, and they include overbreeding, bioremediation, transgenics and developed fertilisation; these techniques have their own limitations of time, ethics and applicability [95]. Using Fe oxides/hydroxides is one of the mitigation techniques to diminish As uptake by plants. Anwar et al. [9] reported that a high rate of goethite addition to soils may decrease As uptake by plants because goethite may adsorb As. Farquhar et al. [96] stated that As(V) oxyanions are toughly ‘sorbed’ to the surfaces of iron oxides such as goethite, hence, As uptake may be reduced by plants. Ultra et al. [92] stated that soil amendments with Am-FeOH can modify the accessibility of As uptake by rice plants irrigated with As-polluted water. The manipulation of Si is a way to abate As uptake by rice. This approach takes advantage of the fact that AsIII, the most prevalent As species in flooded porewater, shares the silicic acid uptake pathway in rice [97]. Liu et al. [98] reported that foliar using SiO_2_ nanoparticles in As-polluted paddy soil increases the dry weight of rice and obviously reduces As accumulation in grains and shoots. The addition of SiO_2_ nanoparticles increases pectin content and advances the mechanical force of the cell wall, resulting in decreased As uptake into rice cells [99]. Rice planted along with accumulators has received growing research attention due to the reduced As uptake by rice. Parveen et al. [100] stated that rice accompanied by accumulators in As-amended plots shows reduced As uptake in grains and shoots. Thus, certain accumulator plants, such as *Pteris vittata*, *Vetiveria zizanioides* and *Phragmites australis*, have been cultivated along with rice. The use of algae or bacteria to reduce As uptake by plants has also been reported [101]. Flooded rice fields offer ideal conditions for microbial and algal growth and regulate As bioavailability through precipitation, redox reactions, complexation and nutrient availability [95].

As shown in Table 3, using microalgae and bacteria are efficient in reducing As accumulation in rice.

## 5. Conclusions

Rice is a major dietary source of As; therefore, researchers have tried to reduce As uptake by rice plants. In the present study, several research papers were reviewed to investigate the journey of arsenic in rice. The key conclusions of the present study are as follows:

1. The accumulation of As in soils in Bangladesh (51,900 µg/L in root) and Taiwan (157,000 µg/L in root) is higher than that in other countries.
2. AsV enters the root via Pi transporters, and AsIII, DMA and MMA enter the root through NIPs.
3. AsV can be reduced to AsIII by HAC1. In addition, DMA is more mobile than other As species.
4. Soil properties, such as pH, OM and the amounts of Fe, Mn, N, P and S, can affect As uptake by rice.
5. Amongst the agronomic strategies for reducing the uptake and accumulation of As in rice, the use of microalgae and bacteria is the most efficient.

## Figures and Tables

**Table 1:** Arsenic found in rice plants around the world.

Plant’s Parts	As (µg/Kg); Average or Range	Remarks	Area	Reference
Grains	230	* Boro rice	Sadar Upazila (subdistrict), Faridpur, Bangladesh	[15]
Straw	2890			
Husk	750			
Grains	235	White rice	Comilla district, Bangladesh	[16]
Straw	1149			
Grains	600	BRRI dhan28	Satkhira district, Bangladesh	[17]
Straw	1700			
Root	46,300			
Grains	700	BRRI hybrid dhan1		
Straw	1900			
Root	51,900			
Grains	78 ± 26	White rice	Barisal, Bangladesh	[18]
	185 ± 82		Chandpur, Bangladesh	
	189 ± 72		Comilla, Bangladesh	
	180 ± 65		Dhaka, Bangladesh	
	177 ± 52		Munshiganj, Bangladesh	
	210 ± 95		Narayanganj, Bangladesh	
Grains	170 to 260	Boro	Bhanga and Faridpur in Bangladesh	[19]
Straw	390 to 3430			
Whole grains	20 to 130	*Oryza sativa* var. kalijira	MATLAB, Bangladesh	
Grains	129.4	White rice	Huang, China	[20]
Grains	250 ± 51	White rice	Renhua, China	[21]
Straw	3300 ± 1300			
Root	25,600 ± 12,500			
Grains	280 ± 67	White rice	Lechang, China	[21]
Straw	5800 ± 2800			
Root	35,000 ± 9400			
Grains	147	Indica	Fujian, China	[22]
	202	Indica	Guangdong, China	
	302	Indica	Guangxi, China	
	200	Indica	Yunnan, China	
	184	Indica	Chongqing, China	
	218	Indica	Sichuan, China	
	187	Japonica	Jiangsu, China	
	277	Indica	Zhejiang, China	
	309	Indica	Jiangxi, China	
	216	Japonica	Henan, China	
	308	Indica	Hunan, China	
	246	Indica	Hubei, China	
	263	Indica	Anhui, China	
	196	Japonica	Liaoning, China	
	426	Japonica	Jilin, China	
		(Unpolished samples)		
Grains	0.127 to 0.275	Indica	Huahang-Simiao, China	[23]
Husk	0.314 to 0.985			
Shoot	0.93 to 6.19			
Root	35.4 to 327.3			
Grains	230 ± 240	Taikeng No. 8	Gaudan Plan, Taiwan	[24]
Straw	4700 ± 1400			
Root	266,000 ± 98,000			
Grain	150 ± 50	Tain Nan No. 11		
Straw	3200 ± 400			
Root	157,000 ± 27,000			
		* Rice Types	Ambagarh Chouki, India	[25]
Husk	432	IR-64		
	147	Culture		
	411	Shyamla		
	415	G. Gurmatia		
	235	Masuri		
	167	Purnima		
	144	Mahamaya		
	446	Kalinga		
	324	Luchai		
	18	Safari		
Grains	3.30 to 4.91	** NR the rice species	Punjab, India	[26]
Straw	7.30 to 9.89			
Grains	451	Boro rice	West Bengal, India	[27]
Grains	334	Aman rice		
Grains	8.78	*Oryza sativa* L.	Central and sub-mountainous Punjab, India	[28]
Straw	3.94			
Grains	290 ± 580	*Oryza sativa*	Alor Setar, Kedah, Malaysia	[29]
Straw	80 ± 150			
Root	23,100 ± 12,670			
Grains	189 to 541	*Oryza sativa*	Besut, Sekinchan, Tanjung Karang and Sabak Bernam; Malaysia	[30]
Grains	124 to 136	Polished rice (White)	Thailand	[31]
	186 to 198	Brown rice (White)		
	832 to 963	Rice bran (White)		
		(Samples collected from markets (Thailand-grown)		
Grains	107 to 166	White rice	Japan; Low-As soils	[32]
Grains				
Grains	160	Brown rice	Japan (average of the country)	[33]
Grains	283 ± 18	* White rice (organic)	Australia (not specified)	[34]
	241 ± 07	White rice (long-grain)		
	438 ± 23	Brown rice (organic)		
	287 ± 03	Brown rice (whole)		
	198 ± 41	Brown rice (long-grain)		
		Samples collected from markets (Australian-grown)		
Grains	170 ± 30	* Mahatma	Australia (not specified)	[35]
	100 ± 30	Brown		
	120 ± 30	White		
	90 ± 20	Medium grain white		
	220 ± 20	Sushi		
	220 ± 20	Arborio		
	210 ± 30	Medium grain Arborio		
		Samples collected from markets (Australian-grown)		
Grains	0.13	*Oryza sativa*	California, US	[36]
Straw	0.7			
Grains	0.2	*Oryza sativa*	Arkansas, US	[36]
Straw	1.5			
Grains	230 ± 10	* Arborio	Lombardia, Piemonte, Emilia Romagna, and Calabria in Italy	[37]
	230 ± 20	Carnaroli		
	180 ± 10	Ribe		
	200 ± 10	Ribe/Roma parboiled		
	190 ± 10	Roma		
	280 ± 30	Vialone Nano		
	190 ± 30	Originario		
Grains	0.32	*Oryza sativa*	Carmargue, France	[36]
Straw	10.2			
Grains	232 ± 21	Brown rice	Guayas, Ecuador	[38]
	174 ± 14	White rice	Guayas, Ecuador	
	186 ± 17	White rice	Los Rios, Ecuador	
		Samples collected from markets (Ecuadorian-grown)		
Grains	167.94		Around Tumbes river basin in Peru	[39]

* Local name. ** NR: Not Reported.

**Table 2:** Gene families involved in As uptake, transport and metabolism in rice.

Name	Category	As species	Remarks	Reference
*OsPT1*	P transporter	AsV	AsV transporter to root	[57]
*OsPT2*	P transporter	AsV	AsV transporter root to shoot	[57]
*OsNIP2;1 (Lsi1)*	NIPs	AsIII, DMA, MMA	AsIII, DMA and MMA transporter to root	[63]
*OsNIP2;2 (Lsi2)*				
*OsNIP1;1*				
*OsNIP3;1*				
*OsNIP3;2*				
*OsNIP3;3*				
*OsPIP1;2*	PIP (plasma membrane intrinsic protein)	AsIII	AsIII transport root to shoot	[65]
*OsPIP1;3*				
*OsPIP2;4*				
*OsPIP2;6*				
*OsPIP2;7*				
*OsNRAMP1*	NRAMP (natural resistance-associated macrophage protein)	AsIII	AsIII transport root to shoot	[5]
*OsHAC1;1*	NIPs	DMA, MMA, AsV	DMA and MMA transporter to root	[63]
*OsHAC1;2*			AsV reduction to AsIII in root	
*OsHAC4*				
*OsNPF8;1 (OsPTR7)*	Putative Peptide Transporter	DMA	Translocation of DMA in plant, including xylem, phloem and grains	[62]
*OsABCC1*	ATP-binding cassette transporter	As	Detoxifying	[66]

**Table 3:** Reported ways for reducing the uptake of As by rice plants.

Decreasing of As Uptake	Method	Remarks	Reference
43% to 70%	Using *Anabaena azotica* (Microalgae)	(i) Decreasing translocation of As from root to grains; (ii) decreasing DMA in grains and roots and (iii) enhancing nutrient uptake and rice growth	[102]
40%	Using *Chlorella vulgaris* and *Nannochloropsis* sp. (Microalgae)	(i) Increasing root and shoot length and biomass and (ii) reduction in cellular toxicity and antioxidant enzyme	[103]
48.1% to 77.7%	Using *Chlorella vulgaris* (Microalgae) and *Pseudomonas putida* (Bacteria)	(i) Reducing As accessibility; (ii) modulating the As uptake and (iii) enhancing detoxification mechanism.	[95]
3.5% to 26.0%	Using rhizobacteria (PGPR)	(i) Improving rice growth and (ii) decreasing As accumulation	[104]
79% (in shoots)	Using *Pantoea* sp (Bacteria; EA106)	(i) improving Fe uptake by root; (ii) decreasing As accumulation	[105]
52.3% to 64.5%	Using *Rhodopseudomonas palustris* C1 and *Rubrivivax benzoatilyticus* C31(Nonsulfur bacteria)	(i) Improving the rice growth; (ii) increasing chlorophyll a and b and (iii) reducing As accumulation	[106]
31% (in grains; just leonardite);	Using leonardite + *Bacillus pumilus*, *Pseudomonas* sp and *Bacillus thuringiensis*	(i) High efficiency of leonardite in adsorption of arsenic and (ii) increasing productivity and reducing arsenic in grains	[107]
92 % (in grains; leonardite + Bacillus pumilus)			
91% (in grains; leonardite + Pseudomonas sp)			
91% (in grains; leonardite + Bacillus thuringiensis)			
17% to 82% (in straw)	Using *Pteris vittata* (Plant)	(i) Decreasing phosphate extractable; (ii) decreasing methylated As in grains more than inorganic As	[108]
22% to 58% (in grains)			
179% (in root)	Using selenium amendments	(i) Enhancing the essential amino acids; and (ii) increasing non-protein thiols and phytochelatins in rice	[109]
144% (in shoot)			
46% (in straw)	Using Si-rich amendments	(i) Decreasing As accumulation and (ii) reducing CH4 emissions from soil	[97]
27.5 (in grains)	Using selenite fertilization	(i) Decreasing the soil solution As in flooded condition; (ii) decreasing As uptake by rice in aerobic and (iii) decreasing the proportion of As in rice shoots.	[110]
50% (straw, flag leaf and husk)	Using silicon	(i) Increasing the Si, Fe and P in soil solution	[111]
68.9% to 78.3% (in grains)	Using ferromanganese oxide and biochar	(i) increasing the Fe and Mn plaque content and (ii) improving the biomass weight of the rice	[112]
32% (in grains under low water)	Using zero valent iron	(i) Increasing percentage productive tillers and grain yield and (ii) reducing the cadmium bioaccumulation in rice grains	[113]
